# A Roadmap for Using Causal Inference and Machine Learning to Personalize Asthma Medication Selection

**DOI:** 10.2196/56572

**Published:** 2024-04-17

**Authors:** Flory L Nkoy, Bryan L Stone, Yue Zhang, Gang Luo

**Affiliations:** 1 Department of Pediatrics University of Utah Salt Lake City, UT United States; 2 Division of Epidemiology Department of Internal Medicine University of Utah Salt Lake City, UT United States; 3 Division of Biostatistics Department of Population Health Sciences University of Utah Salt Lake City, UT United States; 4 Department of Biomedical Informatics and Medical Education University of Washington Seattle, WA United States

**Keywords:** asthma, causal inference, forecasting, machine learning, decision support, drug, drugs, pharmacy, pharmacies, pharmacology, pharmacotherapy, pharmaceutic, pharmaceutics, pharmaceuticals, pharmaceutical, medication, medications, medication selection, respiratory, pulmonary, forecast, ICS, inhaled corticosteroid, inhaler, inhaled, corticosteroid, corticosteroids, artificial intelligence, personalized, customized

## Abstract

Inhaled corticosteroid (ICS) is a mainstay treatment for controlling asthma and preventing exacerbations in patients with persistent asthma. Many types of ICS drugs are used, either alone or in combination with other controller medications. Despite the widespread use of ICSs, asthma control remains suboptimal in many people with asthma. Suboptimal control leads to recurrent exacerbations, causes frequent ER visits and inpatient stays, and is due to multiple factors. One such factor is the inappropriate ICS choice for the patient. While many interventions targeting other factors exist, less attention is given to inappropriate ICS choice. Asthma is a heterogeneous disease with variable underlying inflammations and biomarkers. Up to 50% of people with asthma exhibit some degree of resistance or insensitivity to certain ICSs due to genetic variations in ICS metabolizing enzymes, leading to variable responses to ICSs. Yet, ICS choice, especially in the primary care setting, is often not tailored to the patient’s characteristics. Instead, ICS choice is largely by trial and error and often dictated by insurance reimbursement, organizational prescribing policies, or cost, leading to a one-size-fits-all approach with many patients not achieving optimal control. There is a pressing need for a decision support tool that can predict an effective ICS at the point of care and guide providers to select the ICS that will most likely and quickly ease patient symptoms and improve asthma control. To date, no such tool exists. Predicting which patient will respond well to which ICS is the first step toward developing such a tool. However, no study has predicted ICS response, forming a gap. While the biologic heterogeneity of asthma is vast, few, if any, biomarkers and genotypes can be used to systematically profile all patients with asthma and predict ICS response. As endotyping or genotyping all patients is infeasible, readily available electronic health record data collected during clinical care offer a low-cost, reliable, and more holistic way to profile all patients. In this paper, we point out the need for developing a decision support tool to guide ICS selection and the gap in fulfilling the need. Then we outline an approach to close this gap via creating a machine learning model and applying causal inference to predict a patient’s ICS response in the next year based on the patient’s characteristics. The model uses electronic health record data to characterize all patients and extract patterns that could mirror endotype or genotype. This paper supplies a roadmap for future research, with the eventual goal of shifting asthma care from one-size-fits-all to personalized care, improve outcomes, and save health care resources.

## Introduction

Asthma is a chronic disease characterized by inflammation, narrowing, and hyperactivity of the airways causing shortness of breath, chest tightness, coughing, and wheezing [1]. Asthma affects about 25 million people in the United States [2]. In 2021, there were 9.8 million exacerbations of asthma symptoms (or asthma attacks) leading to over 980,000 emergency room (ER) visits and over 94,500 hospitalizations [2]. Asthma costs the US economy over US $80 billion in health care expenses each year, work and school absenteeism, and deaths [3].

Inhaled corticosteroid (ICS) is a mainstay treatment for controlling asthma and preventing exacerbations in patients with persistent asthma [4] accounting for over 60% of people with asthma [5,6]. Many types of ICS drugs are used, either alone like fluticasone (Flovent, Arnuity, and Aller-flo), budesonide (Pulmicort, Entocort, and Rhinocort), mometasone (Asmanex), beclomethasone (Beclovent, Qvar, Vancenase, Beconase, Vanceril, and Qnasl), ciclesonide (Alvesco), and so forth, or in combination with a long-acting beta2 agonist like fluticasone/salmeterol (Advair), budesonide/formoterol (Symbicort), mometasone/formoterol (Dulera), and fluticasone/vilanterol (Breo), and so forth [4]. Regular use of appropriate ICSs improves asthma control and reduces airway inflammation, symptoms, exacerbations, ER visits, and inpatient stays [7-9].

Despite the widespread use of ICSs, asthma control remains suboptimal in many people with asthma [10-13] including 44% of children and 60% of adults based on asthma exacerbations in the past year [14,15], 72% of patients based on asthma control test [10], 53% of children and 44% of adults based on asthma attacks in the past year [16], and 59% of children based on the 2007-2013 Medical Expenditure Panel Survey [17]. Suboptimal control leads to recurrent exacerbations, causes frequent ER visits and inpatient stays, and is projected to have an economic burden of US $963.5 billion over the next 20 years [18]. Suboptimal control is due to multiple factors [19-23] including (1) failure to recognize and act on early signs of declining control [24,25], (2) lack of self-management skills, (3) nonadherence to therapy [26], and (4) inappropriate ICS choice for the patient [27-32]. While interventions targeting other factors exist, less attention has been given to inappropriate ICS choice.

Asthma is heterogeneous with variable profiles in terms of clinical presentations (phenotypes) and underlying mechanisms (endotypes) [33,34]. Molecular techniques have revealed a few phenotype and endotype relationships, allowing the categorization of asthma into two main groups (1) T-helper type 2 (Th2)-high (eg, atopic and late onset) and (2) Th2-low (eg, nonatopic, smoking-related, and obesity-related) [33,34]. It is known that within the 2 groups, there are many subgroups [33,35] with different biomarker expressions (eg, immunoglobulin E [IgE], fractional exhaled nitric oxide [FeNO], interleukin [IL]-4, IL-5, and IL-13) [36]. So far, only a few biomarkers have been characterized for use in clinical practice. Despite a few successes using biomarkers for targeted therapy, ICS choice, especially in the primary care setting, is largely by trial and error and many patients remain uncontrolled [37-42].

Besides patient nonadherence and environmental factors, response to ICS treatment is affected by genetic variations in ICS metabolizing enzymes [43,44], regardless of whether the ICS is used alone or is combined with another asthma medication like a long-acting beta2 agonist. Single nucleotide polymorphisms in cap methyltransferase 1 (CMTR1), tripartite motif containing 24 (TRIM24), and membrane associated guanylate kinase, WW and PDZ domain containing 2 (MAGI2) genes were found to be associated with variability in asthma exacerbations [43]. Additional evidence supports that these genes also cause variability in ICS response [44]. Due to genetic variations in cytochrome P (CYP) 450 enzymes that metabolize over 80% of drugs including ICS, up to 50% of people with asthma have altered metabolism to certain ICSs [45-51] impacting asthma control [52,53]. CYP3A5*3/*3 and CYP3A4*22 genotypes were found to be linked to ICS response [54,55]. These studies provide evidence that genetic variations greatly affect ICS responsiveness, although the exact relationships between genetic variations and ICS response remain largely unknown [36,56,57]. Currently, many candidate genes are being studied, and pharmacogenetics has not yet reached routine clinical practice in asthma care.

ICS choice for patients is often dictated by insurance reimbursement, organizational policies, or cost, leading to a one-size-fits-all approach [37-42]. Some insurers require patients to first fail on a cheaper ICS before authorizing a more expensive ICS [39]. Nonmedical switch due to preferred drug formulary change is common and leads to bad outcomes, with 70% of patients reporting more exacerbations after the switch [39]. Patients also often report that they tried a few different ICSs before ending up with the drug that gave them the most relief, with 60% reporting it was hard for their providers to find the effective drug [37-39]. Cycling through various ICSs delays the start of an effective ICS and is neither efficient nor cost-effective [39]. New strategies are needed to allow a faster and more efficient way to tailor ICS selection to each patient’s characteristics [36].

While the biologic heterogeneity of asthma is vast, few, if any, biomarkers or genotypes can currently be used to systematically profile all patients with asthma and predict ICS response [36,58,59]. Readily available electronic health record (EHR) data collected during clinical care offer a low-cost, reliable, and more holistic way to profile all patients [36,60]. With a high accuracy of 87%-95% [36], machine learning models using EHR data have been used to profile patients in various areas, for example, to develop a phenotype for patients with Turner syndrome [61], identify low medication adherence profiles [62], find variable COVID-19 treatment response profiles [63], and predict hypertension treatment response [64]. Yet, while machine learning has helped find various asthma profiles [65-72], no prior study has predicted ICS response. Also, prior studies are mostly from single centers with small sample sizes and have not moved the needle of precision treatment for asthma [58,60].

A decision support tool is greatly needed, especially in the primary care setting, to guide providers to select at the point of care the ICS that will most likely and quickly ease patient symptoms and improve asthma control. Forecasting which patient will respond well to which ICS is the first step toward creating this tool, but no prior study has predicted ICS response, forming a gap.

To shift asthma care from one-size-fits-all to personalized care, improve outcomes, and save health care resources, we make three contributions in this paper, supplying a roadmap for future research: (1) we point out the above-mentioned need for creating a decision support tool to guide ICS selection; (2) we point out the above-mentioned gap in fulfilling this need; and (3) to close this gap, we outline an approach to create a machine learning model and apply causal inference to predict a patient’s ICS response in the next year based on the patient’s characteristics. We present the central ideas of this approach in the following sections.

## Creating a Machine Learning Model and Applying Causal Inference to Predict ICS Response

### Overview of Our Approach

We use EHR data from a large health care system to develop a machine learning model and apply casual inference to predict a patient’s ICS response based on the patient’s characteristics. As endotyping or genotyping all patients is infeasible, our model uses EHR data to characterize all patients and extract patterns that could mirror endotype or genotype. Our model is trained on historical data, and can then be applied to new patients to guide ICS selection during an initial or early encounter for asthma care. The optimal ICS choice identified by our approach can be either an ICS (generic name and dosage) alone or an ICS combined with another asthma medication like a long-acting beta2 agonist.

Both pediatric and adult patients with asthma are treated by primary care providers (PCPs) who are mostly generalists and asthma specialists including allergists, immunologists, and pulmonologists. Large differences exist between PCPs and specialists in terms of knowledge, care patterns, and asthma outcomes, with asthma specialists adhering more often to guideline recommendations [73-76]. A greater difference exists between PCPs and specialists in controller medication use [76]. Compared to PCPs, asthma specialists tend to achieve better outcomes [77], including higher physical functioning [78], better patient-reported care [78], and fewer ER visits and inpatient stays [78-84]. As over 60% of people with asthma are cared for by PCPs [85], our machine learning model primarily targets PCPs, although asthma specialists could also benefit from this model.

The asthma medication ratio (AMR) is the total number of units of asthma controller medications dispensed divided by the total number of units of asthma medications (controllers + relievers) dispensed [86,87]. Higher AMR (≥0.5) is associated with less oral corticosteroid use (a surrogate measure for asthma exacerbations), fewer ER visits and inpatient stays, and lower costs [87-89]. Lower AMR (<0.5) is associated with more exacerbations, ER visits, and inpatient stays [90,91]. Approved by Healthcare Effectiveness Data and Information Set (HEDIS) as a quality measure, AMR is widely used by health care systems [89]. AMR is a reliable reflection of asthma control and gives an accurate assessment of asthma exacerbation risk [92]. We use change in AMR as the prediction target of our model for predicting ICS response, as AMR can be calculated on all patients. In comparison, neither asthma control nor acute outcomes (eg, ER visits, inpatient stays, or oral corticosteroid use) is used as the prediction target, as the former is often missing in EHRs and the latter does not occur in all patients. An effective ICS will lead to less reliever use and increased AMR. An ineffective ICS will lead to more reliever use and reduced AMR. We formerly used EHR data to build accurate models to predict hospital use (ER visit or inpatient stay) for asthma [93-95]. We expect EHR data to have great predictive power for AMR, which is associated with hospital use for asthma [87-91]. Using the AMR can facilitate the dissemination of our approach across health care systems.

We outline the individual steps of our approach in the following sections.

### Step 1: Building a Machine Learning Model to Predict a Patient’s ICS Response Defined by Changes in AMR

We focus on patients with persistent asthma for whom ICSs are mainly used. We use the HEDIS case definition of persistent asthma [96,97], the already validated [98] and the most commonly used administrative data marker of persistent asthma [97]. A patient is deemed to have persistent asthma if in each of 2 consecutive years, the patient meets at least one of the following criteria: (1) at least 1 ER visit or inpatient stay with a principal diagnosis code of asthma (*ICD-9* [*International Classification of Diseases, Ninth Revision*] 493.0x, 493.1x, 493.8x, 493.9x; *ICD-10* [*International Classification of Diseases, Tenth Revision*] J45.x), (2) at least 2 asthma medication dispensing and at least 4 outpatient visits, each with a diagnosis code of asthma, and (3) at least 4 asthma medication dispensing. In the rest of this paper, we always use patients with asthma to refer to patients with persistent asthma. The prediction target or outcome is the amount of change in a patient’s AMR after 1 year. The AMR is computed over a 1-year period [86,87].

We combine patient, air quality, and weather features computed on the raw variables to build the model to predict ICS response. Existing predictive models for asthma outcomes [93-95,99-110] rarely use air quality and weather variables, but these variables impact asthma outcomes [111-117] (eg, short-term exposure to air pollution, even if measured at the regional level, is associated with asthma exacerbations [113-117]). For each such variable, we examine multiple features (eg, mean, maximum, SD, and slope). We examine over 200 patient features listed in our papers’ [93-95] appendices and formerly used to predict hospital use for asthma, which is associated with AMR [87-91]. Several examples of these features are comorbidities, allergies, the number of the patient’s asthma-related ER visits in the prior 12 months, the total number of units of systemic corticosteroids ordered for the patient in the prior 12 months, and the number of primary or principal asthma diagnoses of the patient in the prior 12 months. We also use as features the patient’s current AMR computed over the prior 12 months [86,87], the generic name and the dosage of the ICS that the patient currently uses, and those of the long-acting beta2 agonist, leukotriene receptor antagonist, biologic or another asthma medication, if any, that is combined with the ICS.

### Step 2: Conducting Causal Machine Learning to Identify Optimal ICS Choice

Our goal is to integrate machine learning and G-computation to develop a method to estimate the causal effects of various ICS choices on AMR for patients with specific characteristics. This causal machine learning method [118] processes large data sets by capturing complex nonlinear relationships between features, thereby revealing the cause-and-effect relationships between ICS choice and change in AMR. We use the machine learning model built in step 1. Using G-computation [119,120], an imputation-based causal inference method, we estimate the potential effects of hypothetical ICS choices with specific dosages on changes in AMR after 1 year. G-computation builds on the machine learning model of the outcome as a function of ICS indicators, ICS dosages, and other features to predict AMR outcomes under different counterfactual ICS choice scenarios. CIs are estimated through 10,000 bootstrap resampling with replacement [121].

We apply causal machine learning to estimate the impact of ICS choices on patients with specific characteristics by averaging predicted AMR after 1 year for a given ICS and these characteristics across all participants. This estimation is contrasted with the averaged predicted outcome in the absence of any ICS choice. The ICS choice with the highest and statistically significant contrast estimation is identified as the optimal choice for patients with these characteristics. All hypotheses can be tested at a significance level of .05.

### Step 3: Assessing the Impact of Adding External Patient-Reported Asthma Control and ICS Use Adherence Data on the Model’s Predictions

EHRs have limitations regarding patient-reported data with extra predictive power such as asthma control and ICS use adherence. For asthma, asthma control and ICS use adherence are critical variables, as (1) a patient’s asthma control fluctuates over time and drives the provider’s decision to prescribe or adjust ICSs and (2) ICS use adherence impacts the patient’s asthma control and helps assess whether the patient is actually responding to an ICS. However, despite their high predictive power for patient outcomes, these variables are not routinely collected or included in EHRs in clinical practice. At Intermountain Healthcare, the largest health care system in Utah, we pioneered the electronic AsthmaTracker, a mobile health (mHealth) app used weekly to assess, collect, and monitor patients’ asthma control and actual ICS use adherence [122]. Like most patient-reported data, these patient-reported variables have been collected on only a small proportion of patients with asthma. To date, 1380 patients with asthma have used the app and produced about 45,000 records of weekly asthma control scores and ICS use adherence data (eg, the ICS’ name and the number of days an ICS is actually used by the patient in that week). If we train a predictive model using EHR and patient-reported data limited to this small proportion of patients, the model will be inaccurate due to insufficient training data. Yet, for these patients, combining their patient-reported data with the outputs of a model built on all patients’ EHR data can help raise the prediction accuracy for them. To realize this, we propose the first method to combine external patient-reported data available on a small proportion of patients with the outputs of a model built on all patients’ EHR data to raise prediction accuracy for the small proportion of patients while maintaining prediction accuracy for the other patients.

**Figure 1:**
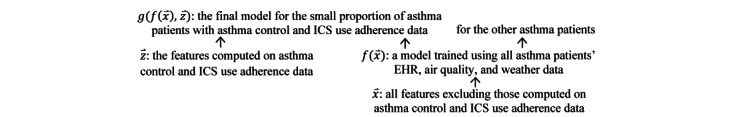
Our method to raise prediction accuracy for the small proportion of patients with asthma and asthma control and ICS use adherence data while maintaining prediction accuracy for the other patients with asthma. EHR: electronic health record; ICS: inhaled corticosteroid.

To illustrate how our method works, we consider the case that the model created in step 1 is built using Intermountain Healthcare EHR data. The weekly asthma control scores and ICS use adherence data collected from the 1380 patients with asthma are unused in step 1. Now we add features (eg, mean, SD, and slope) computed on patient-reported asthma control and ICS use adherence data to raise prediction accuracy for these patients. Among all patients with asthma, only 1% have asthma control and ICS use adherence data. We use the method shown in Figure 1 to combine the asthma control and ICS use adherence data from this small proportion of patients with the outputs of a model trained on EHR, air quality, and weather data of all patients with asthma. We start from the original model built in step 1. This model is reasonably accurate, as it is trained using EHR, air quality, and weather data of all patients with asthma and all features excluding those computed on asthma control and ICS use adherence data. For each patient with asthma control and ICS use adherence data, we apply the model to the patient, obtain a prediction result, and use this result as a feature. We then combine this new feature with the features computed on asthma control and ICS use adherence data to train a second model for these patients using their data. The second model is built upon and thus tends to be more accurate than the original model for these patients. The original model is used for the other patients. Our method is general, works for all kinds of features, and is not limited to any specific disease, prediction target, cohort, or health care system. Whenever a small proportion of patients have extra predictive variables, we could use this method to raise prediction accuracy for these patients while maintaining prediction accuracy for the other patients.

For the patients with asthma control and ICS use adherence data, we compare the mean squared and the mean absolute prediction errors gained by the model built in step 1 and the second model built here. We expect adding asthma control and ICS use adherence data to the model to lower both prediction errors. The error drop rates help reveal the value of routinely collecting asthma control and ICS use adherence data in clinical care to lower prediction errors. Currently, such data are rarely collected.

## Discussion

### Principal Findings

Besides the variables mentioned in the “Step 1: Building a machine learning model to predict a patient’s ICS response defined by changes in AMR” section, environmental variables beyond air quality and weather and many other factors can impact patient outcomes. Moreover, there are almost infinite possible features. For any first future study that one will do along the direction pointed out in this paper, a realistic goal is to show that using our methods can build decent models and improve asthma care rather than to exhaust all possible useful variables and features and obtain the theoretically highest possible model performance. Not accounting for all possible factors limits the generalizability of these models to medication selection for other diseases.

We use the G-computation method to conduct causal inference. This method relies heavily on correctly specifying the predictive model for ICS response, including accurately identifying all relevant confounders and interactions and incorporating them into the model. Misspecification of the model can lead to biased estimated effects of various ICS choices on AMR. To address this issue, we can adopt several preventive strategies during model development. We engage with subject matter experts to ensure that the model includes all relevant variables and reflects the underlying process. To guide model development and help identify potential sources of bias, we construct a directed acyclic graph that lays out the relationships among the independent and dependent variables. We use machine learning techniques that provide flexible modeling approaches to capture complex relationships among variables. When reporting our findings, we keep transparent about the final model specification and the rationale behind our model building process. We believe using these strategies will mitigate the risk of model misspecification and strengthen the reliability of our estimated effects of various ICS choices on AMR.

AMR is reported to be a reliable reflection of asthma control and of asthma exacerbation risk [92]. In a future study that we plan to do along the direction pointed out in this paper, we can use Intermountain Healthcare data to validate this relationship. Specifically, we use multivariable linear regression to assess the relationship between the AMR computed on EHR data and the patient’s asthma control level obtained from the external patient-reported data, while controlling for other factors. We expect to see a strong and positive association between the AMR and the patient’s asthma control level.

When creating the model in step 1, we can include medication persistence measures computed on insurance claim data [123], such as the proportion of days covered for ICS, as features. However, this does not obviate the need to examine patient-reported ICS use adherence data in step 3. ICS persistence measures give information on the possession of ICS, but not on actual use of ICS. Each ICS persistence measure is computed at a coarse time granularity as an average value over a long period. In comparison, our patient-reported ICS use adherence data offer information on the actual use of ICS. The data are at a fine time granularity, with 1 set of values per week for a patient. This enables us to compute features on various patterns and trends that can be useful for making predictions.

### Conclusions

In asthma care, ICS choice is largely by trial and error and often made by a one-size-fits-all approach with many patients not achieving optimal outcomes. In this paper, we point out the need for creating a decision support tool to guide ICS selection and a gap in fulfilling this need. Then we outline an approach to close this gap via creating a machine learning model and applying causal inference to predict a patient’s ICS response in the next year based on the patient’s characteristics. This supplies a roadmap for future research.

## Abbreviations

AMR: asthma medication ratio
CMTR1: cap methyltransferase 1
CYP: cytochrome P
EHR: electronic health record
ER: emergency room
FeNO: fractional exhaled nitric oxide
HEDIS: Healthcare Effectiveness Data and Information Set
*ICD-9*: *International Classification of Diseases, Ninth Revision*
*ICD-10*: *International Classification of Diseases, Tenth Revision*
ICS: inhaled corticosteroid
IgE: immunoglobulin E
IL: interleukin
MAGI2: membrane associated guanylate kinase, WW and PDZ domain containing 2
mHealth: mobile health
PCP: primary care provider
Th2: T-helper type 2
TRIM24: tripartite motif containing 24
